# Physician Perceptions of Oral Health for Pregnant Women: A Bangladesh Perspective

**DOI:** 10.7759/cureus.105602

**Published:** 2026-03-21

**Authors:** Nabhira Aftabi Binte Islam, Taslima Chowdhury, M Atiqul Haque

**Affiliations:** 1 Public Health and Informatics, Bangabandhu Sheikh Mujib Medical University, Dhaka, BGD

**Keywords:** bangladesh, maternal oral care, physician perception, pregnancy complications, pregnant women

## Abstract

Background: This study aimed to investigate obstetricians' knowledge, attitudes, and practices regarding oral health care for pregnant women in Bangladesh. Obstetricians are crucial collaborators with dental surgeons to address oral problems during pregnancy and ensure safe, optimal care.

Methods: A cross-sectional study involving 211 respondents in four divisions of Bangladesh from July 1 to December 19, 2023. Eight medical colleges, comprising an equal number of public and private institutions, were randomly selected to ensure balanced representation. A structured questionnaire consisting of 34 items was developed following an extensive literature review. The tool covered sociodemographic characteristics (7 items) and physicians’ knowledge (12 items), attitudes (7 items), and practices (8 items) regarding oral health care for pregnant women. Spearman correlation coefficients were calculated to examine associations among KAP scores and demographic variables, with significance set at p < 0.05.

Results: Three-quarters of respondents (158, 75%) recognized a connection between hormonal changes and an increased risk of gum disease, while 60 (28.4%) respondents asserted that gum disease is linked to adverse pregnancy outcomes. Moreover, 185 (87.7%) respondents acknowledged a connection between maternal oral health and other health conditions. A total of 184 (87.2%) obstetricians advised brushing twice daily, and 190 (90%) recommended dental consultations for related problems. However, 110 (52%) respondents did not routinely provide oral health advice to expectant mothers. Additionally, 130 (61.6%) participants reported that their undergraduate curriculum did not include pregnancy-related oral health information. Standardized knowledge scores were positively correlated with education (r = 0.189, p < 0.01), age (r = 0.179, p < 0.01), and work experience (r = 0.158, p < 0.05). Standardized attitude scores were positively correlated with age (r = 0.159, p < 0.05) and work experience (r = 0.193, p < 0.01).

Conclusion: Oral health education for obstetricians is essential to improve oral health care for expectant mothers and achieve better health outcomes.

## Introduction

The relationship between pregnancy and oral health is complex due to the significant impact of physiological changes during pregnancy and the connections among hormonal changes, oral health concerns, and their potential consequences on the health of both the mother and the fetus [1,2]. There is a link between poor maternal oral health and unfavourable pregnancy outcomes, such as preterm birth and low birth weight [3,4]. Pregnant women are vulnerable to a wide range of oral health problems, which can affect the health of both the mother and the baby in different ways [5]. There is an association between maternal periodontitis and adverse pregnancy outcomes, and a gynecologist could be the first to assess this [6]. This underscores the pivotal role of medical professionals in encouraging dental treatment among expectant mothers and advocates for culturally tailored oral healthcare guidelines to meet the diverse needs of pregnant women worldwide [7,8].

Understanding these important issues is essential for developing comprehensive antenatal care guidelines [9,10] that address obstetric concerns and oral health considerations. A shortage of knowledge not only affects obstetricians and gynecologists but also leads to neglect, indicating a broader gap in understanding oral healthcare during pregnancy across healthcare professions [11]. In South Asia, such as Bangladesh, pregnant women typically visit their gynecologists or midwives at the onset of pregnancy or when they have concerns during pregnancy. Measuring how often pregnant women visit a healthcare provider during pregnancy is not enough. In one study, 79% of obstetricians and gynecologists in China acknowledged a lack of oral healthcare knowledge during pregnancy [12]. In Brunei Darussalam, approximately 94% of healthcare providers reported that they perceived oral health to be of high importance. However, less than two-thirds of them included oral health questions during antenatal health assessments [1], and only 6% of women visited their dentists during pregnancy, indicating a low rate of oral healthcare utilization during this crucial period. Additionally, 3% of women have never visited a dentist throughout their lives, suggesting that a significant portion of the population lacks regular dental check-ups [13]. It is also important to consider the quality of care they receive, the associated costs, and the impact on their health [14]. By 2030, all nations are expected to have a maternal mortality ratio (MMR) of no more than 70 per 100,000 live births, according to the Sustainable Development Goals [15].

Therefore, integrating oral health into prenatal care is a critical aspect that demands special attention from healthcare professionals, particularly in the maternal healthcare context of Bangladesh. This study aimed to gain insights into obstetricians' knowledge, attitudes, and practices regarding oral healthcare for pregnant women in Bangladesh.

## Materials and methods

Study design and participants

A cross-sectional study was conducted in four administrative divisions of Bangladesh: Dhaka, Mymensingh, Chittagong, and Khulna. The study involved selecting eight medical colleges, four government and four private, with one from each category in each division, between July 1 and December 19, 2023. This selection was made through simple random sampling using all medical colleges in these divisions as the sampling frame. This deliberate choice aimed to ensure a balanced representation of public and private healthcare sectors.

Data were collected through in-person, face-to-face interviews. Participants were purposively selected from the Departments of Obstetrics and Gynecology. To maximize the response rate, researchers approached participants in their respective departments during break hours. To ensure participation remained voluntary and to respect the physicians' professional schedules, individuals who were unavailable or declined to participate were recorded as non-respondents, and no follow-up was conducted. Participants included practicing obstetricians, departmental physicians, and trainees pursuing various postgraduate programs in gynecology and obstetrics.

The primary sample size was determined to be 216, considering that the proportion of participants with appropriate knowledge on maintaining oral health hygiene during pregnancy was 50%, the degree of error was 8%, and the non-response rate was 10%. However, 211 samples were ultimately collected from eight medical institutions across four divisions in Bangladesh.

Tool development and statistical analysis

A structured questionnaire was developed after a thorough review of relevant literature (Appendices). The questionnaire contained 34 items divided into two sections: the first focused on sociodemographic variables such as age, sex, religion, marital status, occupational status, job designation, and duration of job experience (7 questions). The second section addressed physicians' knowledge (12 questions), attitudes (7 questions), and practices (8 questions) regarding oral healthcare for pregnant women. For the knowledge assessment, respondents answered questions with "Yes," "No," or "Don't know." Each correct response ("Yes") was assigned 1 point, while incorrect ("No") or "Don't know" responses were assigned 0 points, resulting in a total knowledge score range of 0 to 12.

Attitudes were assessed using seven items. Responses were initially collected on a four-point Likert scale and subsequently grouped into binary categories: "Strongly disagree/Disagree" (coded as 0) and "Agree/Strongly agree" (coded as 1), resulting in a total attitude score range of 0 to 7.

Practices were assessed using 8 items based on respondents' self-reported behaviors, categorized as "Yes," "Sometimes," and "No," and coded as No (0), Sometimes (1), and Yes (2), with a total score range of 0 to 16.

Before data collection, the questionnaire was pretested among 11 participants from the Gynecology and Obstetrics Department of Bangladesh Medical University, Dhaka, Bangladesh, and necessary adjustments were made based on the feedback received during pretesting.

Categorical data were presented as frequencies and percentages, while continuous data were presented as means and standard deviations (SDs). Knowledge, attitude, and practice scores were standardized by dividing each score by the maximum possible score (standard score = raw score / maximum raw score). The Spearman correlation coefficient was computed to examine relationships among knowledge score, attitude score, practice score, education (0 = no post-graduation, 1 = post-graduation), age, years of experience, and marital status (0 = others, 1 = married). Significance was set at p < 0.05. Statistical analysis was conducted using IBM SPSS Statistics for Windows, Version 20 (Released 2015; IBM Corp., Armonk, New York)

Ethical considerations

Ethical approval was obtained from the Institutional Review Board, Bangabandhu Sheikh Mujib Medical University (reference: 2023/6503). Participants were informed about the study's objectives, assured of confidentiality, and informed that they could withdraw at any time without penalty. Informed consent was obtained from all participants, and written permission was secured from the relevant medical college authorities.

## Results

The mean age of the study participants was 37.6 years (SD ± 9.4). The majority were female (207, 98.1%) and married (184, 87.2%). Almost half of the respondents (106, 50.2%) were employed by the government, while 99 (46.9%) held postgraduate medical degrees. Additionally, 49 (23.2%) of respondents held positions of Associate Professor and above, and 67 (31.8%) reported having more than 10 years of work experience (Table 1).

**Table 1:** Sociodemographic characteristics of the respondents (N = 211) All values are presented as frequencies and percentages unless stated otherwise. MBBS: Bachelor of Medicine and Bachelor of Surgery.

Characteristics	Value
Age in years (mean, SD)	37.6±9.4
Sex	Frequency (%)
Male	4 (1.9)
Female	207 (98.1)
Religion	
Islam	181 (85.8)
Hinduism	27 (12.8)
Buddhism	3 (1.4)
Marital status	
Married	184 (87.2)
Unmarried	27 (12.8)
Occupational status	
Government	106 (50.2)
Non-government	105 (49.8)
Highest degree	
MBBS	112 (53.1)
Post-graduate	99 (46.9)
Current designation	
Assistant Professor and below	162 (76.8)
Associate Professor and above	49 (23.2)
Work experience	
Low experience (1-10 Years)	144 (68.2)
High experience (>10 Years)	67 (31.8)

The total knowledge score among physicians regarding oral health during pregnancy was 6.84 (SD 1.77). Approximately 96 (45.5%) respondents recognized increased gum bleeding, swelling, and redness during pregnancy, whereas 64 (30.3%) denied encountering such issues. Regarding calcium depletion and decay, 141 (66.8%) of respondents believed there was an association, while 46 (21.8%) disagreed. When asked about the relationship between hormonal changes and gum disease, 159 (75.4%) of respondents agreed that hormonal fluctuations during pregnancy contribute to gum disease, with 27 (12.8%) expressing disagreement. Regarding dental X-rays during pregnancy, 127 (60.2%) recommended avoidance, while 74 (35.1%) correctly identified that they are not strictly contraindicated.

Sixty (28.4%) of respondents linked maternal gum disease with adverse pregnancy outcomes, while 91 (43.1%) disagreed. Additionally, 90 (42.7%) agreed that cariogenic bacterial transmission occurs during pregnancy, though 76 (36.0%) respondents did not know this possibility. Regarding concerns about fetal harm from local anesthesia, only 29 (13.7%) of participants believed that local anesthesia could harm the fetus, while a substantial majority of 144 (68.2%) disagreed with this notion. Furthermore, 185 (87.7%) of respondents acknowledged a strong link between maternal oral health and other health conditions, with only 12 (5.7%) denying this relationship. Lastly, while 202 (95.7%) of participants emphasized the importance of maternal oral care, only 81 (38.4%) reported its inclusion in their undergraduate medical curriculum.

Regular oral care during pregnancy is widely emphasized, although fewer physicians reported that oral health information was included in their academic training. There is also a mixed understanding of the safety of fluoride toothpaste and the practice of brushing after vomiting during pregnancy. Overall, the knowledge level among physicians highlights areas for improvement in training and awareness regarding oral health in pregnant women (Table 2). 

**Table 2:** Physician knowledge about oral diseases in pregnant women (N = 211) *Mean score based on binary coding (0 = No/Don't know; 1 = Yes). Total possible score range: 0–12. Total knowledge score: 6.84 ± 1.77.

Knowledge	Yes (%)	No (%)	Don’t know (%)	Mean score*
Importance of regular oral care for mothers	202 (95.7)	6 (2.8)	3 (1.4)	0.96
Maternal oral health connected to other health conditions	185 (87.7)	12 (5.7)	14 (6.6)	0.88
Hormonal changes increase gum disease risk	159 (75.4)	27 (12.8)	25 (11.8)	0.75
Fetal development depletes calcium/causes decay	141 (66.8)	46 (21.8)	24 (11.4)	0.21
Fluoride toothpaste is safe	128 (60.7)	25 (11.8)	58 (27.5)	0.60
Avoid dental X-rays during pregnancy	127 (60.2)	74 (35.1)	10 (4.7)	0.35
Brushing after vomiting is beneficial	100 (47.4)	80 (37.9)	31 (14.7)	0.38
Increased gum bleeding, swelling, and redness	96 (45.5)	64 (30.3)	51 (24.2)	0.69
Cariogenic bacteria pass to the fetus	90 (42.7)	45 (21.3)	76 (36.0)	0.42
Oral health in pregnancy included in the curriculum	81 (38.4)	130 (61.6)	0 (00)	0.62
Gum disease linked to adverse pregnancy outcomes (LBW, premature birth, and preeclampsia)	60 (28.4)	91 (43.1)	60 (28.4)	0.28
Local anesthesia harmful to the fetus	29 (13.7)	144 (68.2)	38 (18.0)	0.68

The total attitude score among physicians regarding oral health during pregnancy was 4.01 (SD 1.43). Most respondents (204, 96.7%) acknowledged the importance of oral health counseling for expectant mothers. Likewise, 202 (95.7%) supported its integration into antenatal care (ANC). Referral to a dental surgeon for oral issues during pregnancy was viewed as important by 197 (93.4%) participants. Additionally, 181 (85.8%) believed dental treatment is safe for pregnant women, while 30 (14.2%) disagreed. Moreover, 195 (92.4%) agreed that oral diseases can be treated safely during pregnancy, while 16 (7.6%) disagreed. Conversely, 185 (87.7%) opposed the avoidance of oral examinations during pregnancy, while 26 (12.3%) agreed with avoiding them. Finally, 169 (80.1%) disagreed with using alternative toothbrushing materials, and 42 (19.9%) favored their use (Table 3).

**Table 3:** Respondents' attitude about oral diseases in pregnant women (N = 211) *Mean score based on binary coding (0 = Disagree/Strongly Disagree; 1 = Agree/Strongly Agree). Total possible score range: 0–7. Total attitude score: 4.01 ± 1.43.

Questions	Strongly disagree (%)	Disagree (%)	Agree (%)	Strongly agree (%)	Mean score (%)*
Importance of oral health counseling for expectant mothers	5 (2.4)	2 (0.9)	109 (51.7)	95 (45.0)	0.97
Oral health counseling in ANC	4 (1.9)	5 (2.4)	117 (55.5)	85 (40.3)	0.96
Referral to the dentist during pregnancy	5 (2.4)	9 (4.3)	95 (45.0)	102 (48.3)	0.93
Safety of dental treatment for pregnant women	5 (2.4)	25(11.8)	129 (61.1)	52 (24.6)	0.86
Safety of treating oral diseases during pregnancy	5 (2.4)	11 (5.2)	139 (65.9)	56 (26.5)	0.92
Avoiding oral exams during pregnancy	77 (36.5)	108 (51.2)	17 (8.1)	9 (4.3)	0.12
Alternative materials for tooth brushing during pregnancy	72(34.1)	97 (46.0)	37 (17.5)	5 (2.4)	0.19

The total practice score among physicians regarding oral health during pregnancy was 4.9 (SD 3.1). The majority of obstetricians (184, 87.2%) advised pregnant women to brush their teeth twice daily, while 16 (7.6%) reported doing so only occasionally. However, providing dental treatment directly was less common, with 126 (59.7%) of providers reporting that they do not offer such care. The recommendation of antibiotics for dental issues also varied: 152 (72.0%) reported they did not advise them, 36 (17.1%) stated they were recommended, and 23 (10.9%) indicated they were sometimes suggested. Notably, 110 (52.1%) healthcare providers reported that they did not routinely offer medical advice related to oral health during pregnancy.

Regarding referrals, 190 (90.0%) of obstetricians advised patients to consult a dentist for tooth and gum issues, while 12 (5.7%) did not. Postnatal dental treatment was recommended by 133 (63.0%) of physicians. Regarding dental X-rays, 130 (61.6%) respondents reported that they advised against them, while 66 (31.3%) stated that they did not give this recommendation. Finally, advice on brushing teeth after vomiting varied: 114 (54.0%) did not provide such advice, 77 (36.5%) provided it, and 20 (9.5%) reported doing so sometimes (Table 4).

**Table 4:** Respondents practice about oral diseases in pregnant women (N = 211) *The mean score was calculated on a 3-point scale where 0 = No, 1 = Sometimes and 2 = Yes. Total possible score range: 0–16. Total practice score: 4.9 ± 3.1.

Questions	No (%)	Yes (%)	Sometimes (%)	Mean score*
Advised to brush teeth twice daily	11 (5.2)	184 (87.2)	16 (7.6)	1.82
Dental treatment provided by gynaecologists and obstetricians	126 (59.7)	48 (22.7)	37 (17.6)	0.63
Antibiotics recommended for dental issues	152 (72.0)	36 (17.1)	23 (10.9)	0.45
No advice was given on oral health	110 (52.1)	49 (23.2)	52 (24.6)	0.71
Advised to consult dentist for tooth/gum issues	12 (5.7)	190 (90.0)	9 (4.3)	1.84
Advised to undergo postnatal dental treatment	37 (17.5)	133 (63.0)	41 (19.4)	1.46
Dental X-rays are not recommended during pregnancy	66 (31.3)	130 (61.6)	15 (7.1)	1.30
Advised to brush teeth after vomiting	114 (54.0)	77 (36.5)	20 (9.5)	0.82

Spearman's rank correlation analysis (Table 5) was performed to examine the relationships between demographic variables and KAP scores. The results showed that the level of education positively correlated with age (r = 0.766, p < 0.001) and work experience (r = 0.733, p < 0.001). Age and work experience were also strongly associated (r = 0.863, p < 0.001). The Standardized Knowledge Score (SKS) was significantly associated with education (r = 0.189, p < 0.01), age (r = 0.179, p < 0.05), work experience (r = 0.158, p < 0.05), and type of job (r = 0.144, p < 0.05). The Standardized Attitude Score (SAS) showed a significant positive correlation with age (r = 0.159, p < 0.05), work experience (r = 0.193, p < 0.01), and SKS (r = 0.137, p < 0.05). However, the Standardized Practice Score (SPS) showed no significant correlations with any demographic variables or other KAP scores. Marital status and type of job were positively associated with education, age, and work experience (all p < 0.001).

**Table 5:** Spearman correlation table between socio-demographic variables and KAP scores (N = 211) ** p < 0.001, * p < 0.05. SKS: Standardized Knowledge Score, SAS: Standardized Attitude Scale, SPS: Standardized Practice Scale.

Attributes	1	2	3	4	5	6	7	8
1. Level of education	1	1	1	1	1	1	1	1
2. Age	.766^**^	1	1	1	1	1	1	1
3. Work-experience	.733^**^	.863^**^	1	1	1	1	1	1
4. SKS	.189^**^	.179^**^	.158^*^	1	1	1	1	1
5. SAS	.095	.159^*^	.193^**^	.137^*^	1	1	1	1
6. SPS	-.056	-.021	-.098	.080	.105	1	1	1
7. Marital status	.303^**^	.370^**^	.332^**^	.073	-.035	-.101	1	1
8. Type of job	.328^**^	.426^**^	.362^**^	.144^*^	-.045	-.085	.357^**^	1

## Discussion

In Bangladesh, pregnant women commonly attend health checkups by registered physicians, particularly obstetricians, or at antenatal care (ANC) units within the Obstetrics and Gynecology departments of secondary and tertiary care hospitals. At the primary healthcare level, in Upazila Health centers, a single obstetric consultant [16] is typically responsible for antenatal and other gynecological care, although general physicians and various non-physician personnel also play a role in delivering these services at the community level. Consequently, obstetricians must be well informed about oral health issues related to pregnancy. However, the present study indicates a relatively low knowledge score regarding oral diseases during pregnancy among physicians, which was reported as 6.84. One of the primary contributors to this lower level of knowledge is the lack of integration of oral health topics into medical curricula [17]. In Bangladesh, both undergraduate and postgraduate medical programs focus extensively on clinical aspects of pregnancy but neglect oral health [18-20]. Approximately 62% of respondents identified this as a critical exclusion in this study.

This study reveals that only 28.4% of physicians lacked knowledge about the association between oral health and adverse pregnancy outcomes such as low birth weight, premature birth, and preeclampsia. Similarly, a study conducted in India found that 37% of physicians were unaware of this link, and only 49% of obstetricians recommended fluoridated toothpaste for gum bleeding during pregnancy [21].

Antenatal care (ANC) in Bangladesh primarily focuses on the general health of pregnant women, with oral health often overlooked, even in updated guidelines implemented in 2019-2030 [22]. In contrast, the ANC guidelines of Bhutan, a neighboring country, incorporate oral health into general health monitoring protocols, highlighting the importance of oral health hygiene during pregnancy [23].

In India, ANC guidelines recommend examining pregnant women for pallor by assessing their conjunctiva, nails, tongue, oral mucosa, and palms to identify anemia. Regular monitoring of oral health conditions is emphasized to enable early detection of complications that could otherwise impact overall maternal health. Despite these recommendations, pregnant women often encounter challenges in accessing oral health guidelines and obtaining necessary treatments [24].

While 97% of obstetricians agreed on the importance of providing oral health counseling during pregnancy, and 96% of respondents supported integrating oral health counseling into ANC in the present study, underscoring its significance in maternal healthcare, only 38.4% reported receiving formal education on oral health in pregnancy. A substantial proportion of healthcare providers (52.1%) did not offer advice on oral health issues. Despite this, 90% of respondents advised pregnant women to consult a dentist, reflecting an awareness of the importance of oral health but a lack of confidence or resources to address it directly.

This study highlighted a conservative approach among respondents, with 60.2% advising against dental X-rays during pregnancy. In contrast, the American Dental Association (ADA) emphasizes that dental radiographs can be safely performed at any stage of pregnancy, provided that appropriate radiation protection measures are implemented to minimize exposure [25]. A lack of appropriate knowledge as well as practice regarding the safety of dental imaging frequently deters pregnant women from seeking necessary dental care. Standardized, evidence-based guidelines and education for healthcare providers are essential to ensure pregnant women receive comprehensive dental care, benefiting both maternal and fetal health.

In the current study, 13.7% of respondents believed that local anesthesia during pregnancy could harm the fetus. However, the study also revealed that local anesthetics are safe at any stage of pregnancy when proper precautions are taken. They emphasize that necessary dental care should not be delayed due to pregnancy [12].

Despite this empirical evidence, some healthcare providers remain unaware of the safety of dental procedures during pregnancy, leading to inconsistent practices and unnecessary delays in treatment. For example, the importance of educating healthcare providers to improve their understanding of the safe and effective use of local anesthetics in pregnant patients [26] was highlighted in one study. This gap in awareness underscores the need for standardized, evidence-based guidelines to ensure consistent and comprehensive maternal care. Addressing these misconceptions is critical to reducing variability in practice and ensuring pregnant women receive timely and appropriate dental treatment.

Integrating oral health measures into ANC guidelines is essential for improving maternal health outcomes. Clear and comprehensive guidelines on safe oral health practices during pregnancy are urgently needed. Many expectant mothers receive inadequate dental care advice, and a substantial number are not advised or offered necessary dental treatments. Addressing this gap can significantly enhance maternal and fetal health outcomes.

The study highlights links between demographic and professional characteristics and knowledge, attitudes, and practices. Higher levels of education, age, and work experience were associated with better knowledge scores (SKS) and more favorable attitudes (SAS) toward oral health in pregnancy. This aligns with other studies that emphasize the role of experience and education in improving healthcare standards [11,27]. However, some variables, such as SPS, showed no significant correlation, suggesting areas for further investigation.

Limitations

Data were collected from only four divisions; including all eight divisions of Bangladesh would provide a more comprehensive national picture. In addition, data collection used self-reported questionnaires, which may lead to social desirability bias, where doctors respond based on what they should do rather than their actual daily practice. Finally, the lack of correlation in practice scores (SPS) suggests that external factors, such as high patient loads, institutional barriers, or a lack of clear referral guidelines, may influence how oral health is managed in antenatal care.

## Conclusions

In Bangladesh, gynaecologists' perceptions of oral health for pregnant women shed light on dental care practices during pregnancy. While many advise regular tooth brushing and dental consultations, inconsistencies exist regarding the provision of dental treatment by obstetricians and guidance on issues such as dental X-rays and postnatal care. Standardized guidelines and evidence-based education are vital for optimal oral health practices among pregnant women. Bridging gaps in the understanding and practice of dental care during pregnancy can improve maternal and pregnancy outcomes. Collaboration between the dental and obstetric sectors is essential for integrating oral health into routine ANC in Bangladesh. These insights underscore the need for consistent dental care recommendations and highlight the importance of research and education in maternal oral health to promote healthier pregnancies.

The following recommendations are proposed. To ensure physicians are well informed about the importance of oral health during pregnancy, oral health training programs should be integrated into their education and practice. Additionally, standardized referral and treatment protocols between obstetricians and dentists should be developed to ensure that pregnant women receive appropriate and timely oral healthcare.

## Appendices

**Table 6:** Initial documents Name of Interviewer: Time:

Initial documents		
Division		
District		
Upzila		
Type of job	Governments	1
	Private	2
	Others	3

**Table 7:** Section A: Sociodemographic information Please read the following statements and put a ‘√’ mark on the option that applies to you.

Question	Response	
Date of Birth	………… Age	
Sex	Male	1
	Female	2
Religion	Islam	1
	Hindu	2
	Christian	3
	Buddhist	4
	Other	5
Marital status	Married	1
	Unmarried	2
	Other	3
What is your highest educational qualification?	MBBS	1
	Post-graduation	2
What is your current designation?	…………	
How many years of working experience do you have in the Gynecology and Obstetrics department?	………… year	

**Table 8:** Section B: Knowledge Please put a "√" mark where applicable.

Question	Options	
Should mothers take regular oral and dental care seriously during pregnancy?	No	0
	Yes	1
	Do not know	2
Is there a connection between maternal oral health and other health conditions during pregnancy?	No	0
	Yes	1
	Do not know	2
Hormonal changes during pregnancy increase the risk of gum disease (Periodontal diseases)?	No	0
	Yes	1
	Do not know	2
Can a developing fetus in the womb deplete the mother's teeth of calcium / cause decay?	No	0
	Yes	1
	Do not know	2
Is it safe for mothers to use fluoride toothpaste during pregnancy?	No	0
	Yes	1
	Do not know	2
Should dental X-rays be avoided during pregnancy?	No	0
	Yes	1
	Do not know	2
Is it better to brush your teeth after vomiting during pregnancy?	No	0
	Yes	1
	Do not know	2
What is the tendency to bleed, swell, or become red during pregnancy?	No	0
	Yes	1
	Do not know	2
During pregnancy, do cariogenic bacteria enter the body of the baby from the mother's body?	No	0
	Yes	1
	Do not know	2
Did your academic curriculum include information on oral health during pregnancy?	No	0
	Yes	1
	Do not know	2
Is a mother's gum disease associated with low birth weight (LBW), premature delivery, and preeclampsia?	No	0
	Yes	1
	Do not know	2
Does local anesthesia in the mouth harm the baby?	No	0
	Yes	1
	Do not know	2

**Table 9:** Section B: Attitude Please put a "√" mark where applicable.

Questions	Strongly disagree	Disagree	Agree	Strongly agree
Oral health counseling of expectant mothers is important				
Oral health counseling should be a part of antenatal care (ANC).				
Referral is important to the dentist for dental treatment during pregnancy				
Dental treatment is safe for pregnant women				
Oral diseases can be treated safely during pregnancy without causing any harm to the unborn baby.				
Oral examination of mothers should not be done during pregnancy.				
Other materials such as salt, mustard oil, toothpaste, charcoal, etc. can be used for tooth brushing during pregnancy.				

**Table 10:** Section B: Practice Please put a "√" mark where applicable.

Question	Response	
Pregnant mothers are advised to brush their teeth twice daily (in 24 hours).	No	0
	Yes	1
	Sometimes	2
Usually, expectant mothers are given dental treatment by the gynecologists themselves	No	0
	Yes	1
	Sometimes	2
Antibiotics are recommended for any dental problem by the gynecologists themselves. For example, bleeding gums, swollen gums, etc.	No	0
	Yes	1
	Sometimes	2
We generally do not give medical or health advice on mouth, teeth, and gum problems for expectant mothers.	No	0
	Yes	1
	Sometimes	2
Pregnant mothers are advised to consult a dentist for tooth and gum problems	No	0
	Yes	1
	Sometimes	2
Pregnant mothers are advised to undergo postnatal dental treatment	No	0
	Yes	1
	Sometimes	2
Dental X-rays are not recommended during pregnancy	No	0
	Yes	1
	Sometimes	2
Pregnant mothers are advised to brush their teeth immediately after vomiting	No	0
	Yes	1
	Sometimes	2
